# Endorsements

**Published:** 1881-07

**Authors:** 


*Endorsements* are good and helpful, and ought to be cheerfully given when deserved, but to read the circulars advertising the various preparations in use, with the highly colored testimonials accompanying them, one almost comes to the conclusion that in some cases either the parties giving the testimonials are paid for them, or else that they are given for the sake of seeing the writer's name in print. One writer says authoritatively, that "your teeth are the best in the market," another that "your preparation of zinc filling is the only one fit for use"—this conclusion is arrived at after six weeks use of it. Another writes that "your amalgam is the only thing that will preserve badly decayed teeth," and so on to the end of the chapter.

Is it possible that the writers of these testimonials really have "tested" these things? Is one year or two years sufficiently long to decide the question whether or not *any* filling will preserve a tooth? Is the mere handling of a preparation and inserting it in a tooth any *test* of its tooth-preserving qualities? And surely the mere leaving with a dentist, by a travelling agent, of a sample of amalgam or other filling, should hardly be a sufficient inducement for the straightway asking for and granting of a certificate of merit. We feel satisfied that in most of the cases in which testimonials are given it is because they are *asked for*, rather than that the writers have accurate knowledge of what they are certifying to.

